# Determination of Incidence and Risk Factors of Hypotension Following Spinal Anesthesia During Cesarean Delivery in Patients With Preeclampsia: A Prospective Observational Study

**DOI:** 10.7759/cureus.110097

**Published:** 2026-06-02

**Authors:** Sandhiya Pannirselvam, Mohan Vasuki Krishnan, Vasudevan Arumugam, Thirumurugan Arikrishnan, Deepak Chakravarthy, Arivarasan Barathi

**Affiliations:** 1 Anaesthesiology, Sri Manakula Vinayagar Medical College and Hospital, Puducherry, IND; 2 Anaesthesiology, Jawaharlal Institute of Postgraduate Medical Education and Research, Puducherry, IND; 3 Anaesthesiology and Critical Care, Indira Gandhi Medical College and Research Institute, Puducherry, IND; 4 Community Medicine, Employees State Insurance Corporation (ESIC) Medical College and Hospital, Chennai, IND

**Keywords:** anesthesia spinal, cesarean section (cs), preeclampsia (pe), risk factor approach, spinal hypotension

## Abstract

Background

Preeclampsia is associated with high-risk pregnancies and significant maternal morbidity. Although the incidence of hypotension is lower in preeclamptic women, its occurrence can lead to adverse maternal outcomes due to altered vascular physiology. This study aimed to determine the incidence of hypotension following spinal anesthesia in preeclamptic women and to identify associated demographic, obstetric, and clinical risk factors.

Methods

This prospective observational study included 180 preeclamptic women undergoing cesarean section under spinal anesthesia. Hypotension was defined as systolic blood pressure <100 mmHg. Univariate logistic regression was performed to identify potential risk factors, and variables with p<0.20 were included in multivariable logistic regression to adjust for confounding.

Results

The incidence of hypotension was 28.33% (51/180). On univariate analysis, early-onset preeclampsia (≤34 weeks), multiple gestation, and lower hemoglobin levels were associated with increased risk of hypotension. In multivariable analysis, anemia remained an independent predictor. Women with hemoglobin 7-9.9 g/dl had 3.15 times higher odds (95% CI: 1.33-7.45; p=0.009), and those with hemoglobin 10-10.9 g/dl had 2.44 times higher odds (95% CI: 1.02-5.84; p=0.044) of hypotension compared to those with hemoglobin >11 g/dl. Early-onset preeclampsia also showed increased odds (AOR 1.71; 95% CI: 0.82-3.56), without statistical significance.

Conclusion

Hypotension following spinal anesthesia occurs in a substantial proportion of preeclamptic women. Optimization of hemoglobin levels and careful perioperative monitoring may help reduce the risk of hypotension and improve maternal outcomes.

## Introduction

Hypertensive disorders of pregnancy (HDP) are a major contributor to maternal and neonatal morbidity and mortality worldwide, complicating nearly 10% of all pregnancies [1]. Among these, preeclampsia remains the most common and clinically significant condition, affecting approximately 5-7% of pregnancies globally [2,3]. It is a multisystem disorder characterized by new-onset hypertension and end-organ dysfunction after 20 weeks of gestation and continues to be one of the leading causes of maternal mortality, particularly in low- and middle-income countries.

The pathophysiology of preeclampsia is complex and not yet fully understood; however, abnormal placentation is widely accepted as the central mechanism. Inadequate trophoblastic invasion of the spiral arteries leads to incomplete vascular remodeling, resulting in high-resistance uteroplacental circulation and reduced placental perfusion. This hypoperfused placenta releases antiangiogenic factors, such as soluble fms-like tyrosine kinase-1 (sFlt-1) and soluble endoglin, which antagonize proangiogenic factors, including vascular endothelial growth factor (VEGF) and placental growth factor (PlGF). The resultant endothelial dysfunction contributes to systemic manifestations, including hypertension, proteinuria, coagulopathy, cerebral edema, and hepatic involvement [3].

The definitive management of preeclampsia is delivery of the fetus and placenta, as no curative medical therapy exists [3]. While expectant management may be considered in selected cases, induction of labor is generally recommended at or beyond 37 weeks of gestation to reduce maternal and fetal complications. However, due to disease severity and obstetric indications, cesarean section is frequently performed in preeclamptic women.

The anesthetic management of these patients poses a significant perioperative challenge. Preeclampsia is associated with multiple physiological alterations, including endothelial dysfunction, reduced intravascular volume, increased systemic vascular resistance, and potential airway edema, all of which complicate anesthetic decision-making [4]. General anesthesia is often considered less desirable in these patients due to the increased risk of difficult airway management, exaggerated hypertensive response to laryngoscopy, and potential drug interactions, particularly with magnesium sulfate, which may prolong neuromuscular blockade and affect uterine tone [5].

Consequently, regional anesthesia, particularly spinal anesthesia, is widely regarded as the preferred technique for cesarean section in women with preeclampsia [6,7]. It offers several advantages, including avoidance of airway manipulation and attenuation of the stress response associated with intubation. Despite these benefits, spinal anesthesia is not without risks, with hypotension being the most significant concern.

Although the incidence of hypotension following spinal anesthesia is reported to be lower in preeclamptic women compared to normotensive pregnant women, transient episodes of hypotension can still occur and may have significant clinical implications [8]. The altered hemodynamic state in preeclampsia, characterized by vasoconstriction and reduced plasma volume, makes these patients particularly vulnerable to reductions in systemic vascular resistance following spinal anesthesia. Importantly, uteroplacental perfusion is not autoregulated, and any fall in maternal blood pressure can lead to compromised fetal oxygenation.

Given these concerns, prevention and early identification of hypotension remain critical in the anesthetic management of preeclamptic patients. While several studies have identified risk factors for spinal-induced hypotension in normotensive pregnant women, including higher body mass index (BMI), multiparity, advanced maternal age, and higher sensory block levels, evidence specific to preeclamptic populations, particularly in regional settings, is limited [9,10].

Therefore, this study aims to identify the risk factors associated with hypotension following spinal anesthesia in preeclamptic women undergoing cesarean section. Understanding these factors will aid in optimizing perioperative management, guiding anesthetic technique selection, and improving maternal and fetal outcomes.

## Materials and methods

Study design and setting

A prospective observational study was conducted to identify the risk factors associated with hypotension following spinal anesthesia in preeclamptic women undergoing cesarean section. The study was conducted at a tertiary care teaching hospital after obtaining approval from the Institutional Ethics Committee (JIP/IEC/2019/445) and registration with the Clinical Trials Registry of India (CTRI/2020/08/027178). The study adhered to ethical principles outlined in the Declaration of Helsinki. Written informed consent was obtained from all participants prior to enrollment.

Study participants

The study population consisted of pregnant women diagnosed with preeclampsia who were scheduled to undergo cesarean section under spinal anesthesia. Eligible participants were recruited consecutively during the study period.

Inclusion criteria included all women diagnosed with preeclampsia undergoing cesarean section under spinal anesthesia. Exclusion criteria comprised women with chronic hypertension, known hypersensitivity to anesthetic agents used in the procedure, and contraindications to spinal anesthesia, such as patient refusal, localized infection at the puncture site, coagulopathy, or inability to maintain a steady position during the procedure. Withdrawal criteria were defined a priori. Participants who required conversion to general anesthesia after administration of spinal anesthesia were excluded from the final analysis.

Sampling and sample size

The sampling population included all eligible preeclamptic women undergoing cesarean section under spinal anesthesia during the study period. A consecutive sampling technique was adopted to minimize selection bias and ensure representativeness.

The sample size was calculated using the formula for the estimation of a single proportion. Based on previous evidence [3], the expected proportion of hypotension following spinal anesthesia in preeclamptic women was assumed to be 26%. With an absolute precision of 5% and a confidence level of 95%, the minimum required sample size was estimated to be 296 participants. Due to the COVID pandemic, the expected admissions to our tertiary care center reduced, and we could not enroll the calculated sample size of 296. A total of 180 preeclamptic women were assessed for determining the incidence and risk factors of hypotension after spinal anesthesia for cesarean section.

Study procedure

All eligible participants were evaluated preoperatively, and baseline demographic and clinical data were recorded. Upon arrival in the operating theater, standard monitoring was initiated, including pulse oximetry, non-invasive blood pressure measurement, and electrocardiography. Baseline hemodynamic parameters, including systolic and diastolic blood pressure and heart rate, were documented prior to the administration of anesthesia.

The choice of premedication, spinal anesthetic drug, and technique was left to the discretion of the attending anesthesiologist, reflecting routine clinical practice. Under strict aseptic precautions, spinal anesthesia was administered in the sitting or lateral position. Following confirmation of adequate sensory blockade to a level between T4 and T6, the surgical procedure was initiated.

Hemodynamic parameters, including systolic and diastolic blood pressure and heart rate, were monitored at five-minute intervals throughout the intraoperative period, in accordance with the routine monitoring practice at the institution during the study period. Hypotensive episodes occurring between monitoring intervals were managed based on clinical assessment and standard anesthetic care.

**Figure 1:**
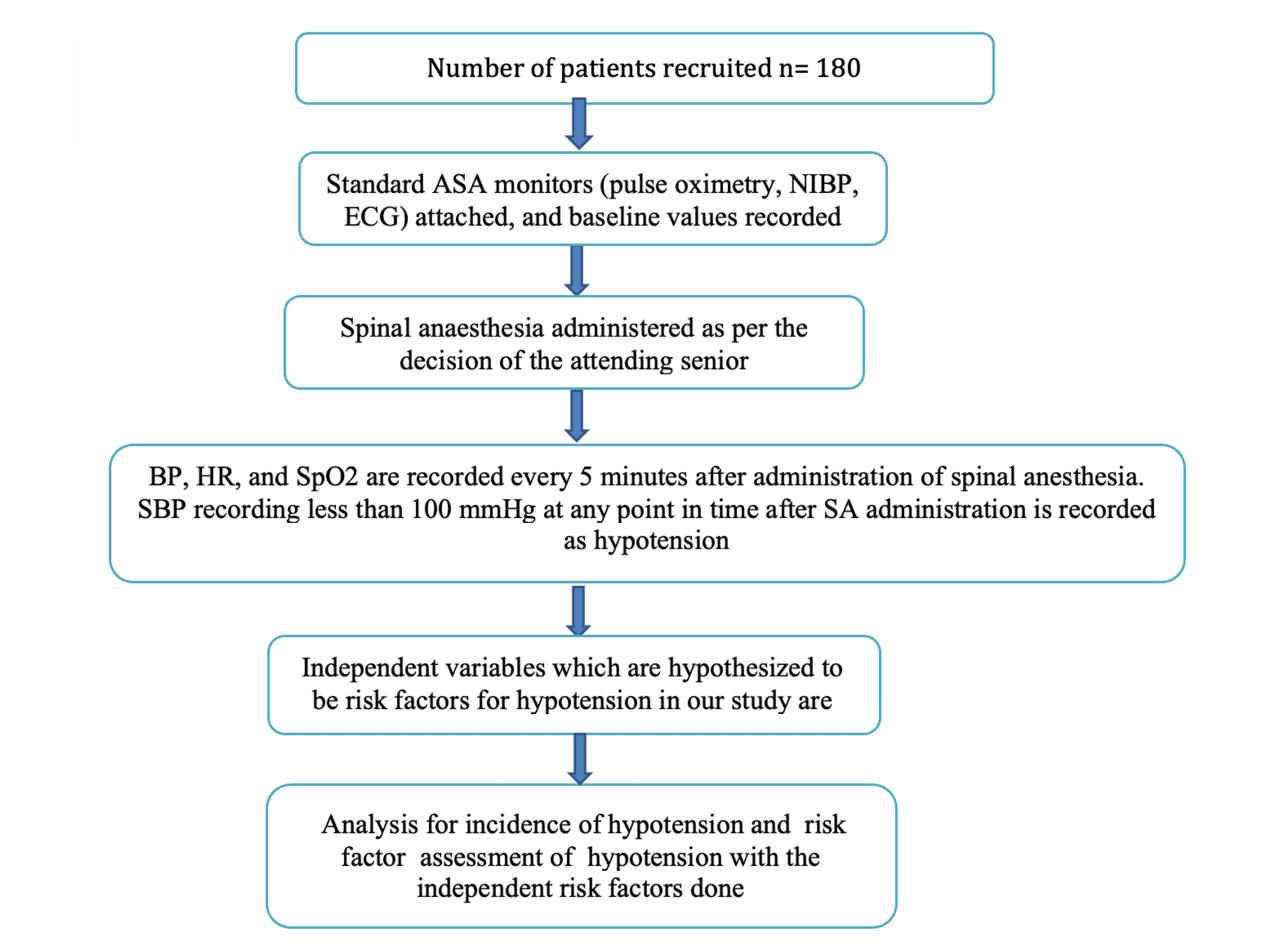
Flowchart of the study ASA: American Society of Anesthesiologists; NIBP: Non-invasive blood pressure; ECG: Electrocardiography; BP: Blood pressure; HR: Heart rate; SpO₂: Peripheral oxygen saturation; SBP: Systolic blood pressure; SA: Spinal anesthesia. The image was created using Microsoft Word (Microsoft Corporation, Redmond, Washington, USA).

Hypotension was operationally defined as a systolic blood pressure of less than 100 mmHg. The primary outcome variable was the occurrence of hypotension, defined as a systolic blood pressure of less than 100 mmHg during the intraoperative period. Although several contemporary studies define spinal anesthesia-induced hypotension as a reduction in systolic blood pressure of 20-30% from baseline, an absolute systolic blood pressure threshold of <100 mmHg has also been used in previous obstetric anesthesia studies and was selected a priori for this study to ensure a clinically uniform and easily applicable definition across all participants. Appropriate management of hypotension, including fluid administration and vasopressor use, was carried out as per standard institutional protocols. The study procedure is detailed in Figure 1.

Data on maternal characteristics, such as age, height, weight, body mass index (BMI), obstetric history, gestational age at the onset of preeclampsia, duration of disease, drug therapy, and preoperative fasting status, were recorded. Intraoperative variables, including fluid administration, estimated blood loss, oxytocin dosage, and vasopressor requirements, were also documented. Neonatal outcomes were assessed using the Apgar score at one and five minutes after delivery.

The institutional protocol for management of spinal anesthesia-induced hypotension included prompt administration of intravenous crystalloid fluids, left uterine displacement, supplemental oxygen when indicated, and vasopressor therapy at the discretion of the attending anesthesiologist. Vasopressor management primarily consisted of intravenous boluses of mephentermine or phenylephrine, depending on availability and clinical judgment. Additional supportive measures were undertaken based on maternal hemodynamic status and fetal well-being.

Variables and measurements

The independent variables included demographic factors (age, height, weight, BMI), obstetric characteristics (gravidity, gestational age at onset and duration of preeclampsia), clinical variables (drug therapy, comorbidities), and intraoperative factors (fluid administration, blood loss, oxytocin dose, vasopressor use, and duration of labor).

The primary outcome variable was the occurrence of hypotension, defined as a systolic blood pressure of less than 100 mmHg during the intraoperative period. Secondary outcome measures included the number of hypotensive episodes, maximum percentage fall in systolic blood pressure from baseline, time interval from spinal anesthesia to the onset of hypotension, and neonatal Apgar scores.

Statistical analysis

Data were entered into Microsoft Excel (Microsoft Corporation, Redmond, Washington) and analyzed using STATA version 14 (StataCorp LLC, College Station, Texas, USA). Categorical variables were summarized as frequencies and percentages, while continuous variables were described using appropriate measures of central tendency and dispersion.

To identify factors associated with hypotension, univariate logistic regression analysis was initially performed for each independent variable. Variables with a p-value less than 0.20 in the univariate analysis were considered for inclusion in the multivariable logistic regression model. Multivariable logistic regression was then performed to adjust for potential confounders and to identify independent predictors of hypotension. Results were expressed as odds ratios (OR) with 95% confidence intervals (CI), and a p-value of less than 0.05 was considered statistically significant.

## Results

Among the 180 preeclamptic women observed in this study, 51 of the preeclamptic women had systolic blood pressure recordings of less than 100 mmHg in the intraoperative period. Thus, the incidence of hypotension observed in our study is 28.33%. Table 1 shows the mean (SD) for age, weight, height, and BMI among 180 women. The mean age among the study subjects was 27.73±5.45 years, weight 69±4.13 kg, height 157.81±2.91 cm, and BMI 27.72±1.48 kg/m².

**Table 1:** Age and anthropometric details of the study participants (n=180)

Parameters	Mean + SD
Age (years)	27.73 (5.45)
Weight (kg)	69 (4.13)
Height (cm)	157.81 (2.91)
Body mass index (kg/m^2^)	27.72 (1.48)

Hypotension following spinal anesthesia was observed in 51 out of 180 participants (28.3%). Among demographic factors, women aged >35 years showed a similar proportion of hypotension (6/20 (30.0%)) compared to those aged ≤35 years (45/160 (28.1%)), without a statistically significant association (OR 1.10, p=0.861). Women with a BMI ≥30 kg/m² demonstrated a higher occurrence of hypotension (8/15 (53.3%)) compared to those with a normal BMI (2/7 (28.5%)), although the association was not statistically significant (OR 2.86, p=0.286). Gravidity also did not show a significant association with hypotension, with proportions comparable across G1 (29/97 (29.9%)), G2 (12/53 (22.6%)), and G3 (6/15 (40.0%)).

Among obstetric factors, multiple gestation pregnancies had a significantly higher proportion of hypotension (5/8 (62.5%)) compared to singleton pregnancies (46/172 (26.7%)) (OR 4.56, p=0.043). Regarding indications for cesarean section, failed induction was associated with a higher incidence of hypotension (5/8 (62.5%)) compared to fetal distress (30/123 (24.4%)) (OR 5.17, p=0.031). Similarly, women with a duration of labor greater than eight hours showed a higher frequency of hypotension (26/67 (38.8%)) compared to those with a labor duration less than four hours (2/17 (11.8%)) (OR 4.76, p=0.049) (Table 2).

**Table 2:** Univariate analysis – patient demographic profile and obstetric profile factors for association with risk of hypotension after spinal anesthesia (N=180) *Variables with a p-value less than 0.20 in the univariate analysis will be taken for multivariate analysis for association with hypotension. BMI: Body mass index; G: Gravida; IVF: In vitro fertilization; LSCS: Lower segment cesarean section; CPD: Cephalopelvic disproportion; SBP: Systolic blood pressure; OR: Odds ratio; CI: Confidence interval.

Characteristics		SBP < 100 n (%) n = 51	SBP ≥100 n (%) n = 129	Odds ratio (OR)	95% Confidence interval (CI)	p-value
Age	>35 years	6 (30.0%)	14 (70.0%)	1.1	0.40–3.03	0.861
	=35 years	45 (28.1%)	115 (71.9%)	Reference	-	-
BMI	18.5–24.9	2 (28.5%)	5 (71.5%)	Reference	-	-
	25.0–29.9	41 (25.95%)	117 (74.05%)	0.876	0.16–4.69	0.877
	30.0–34.9	8 (53.33%)	7 (46.67%)	2.857	0.41–19.64	0.286
Gravidarum	G1	29 (29.90%)	68 (70.10%)	Reference	-	-
	G2	12 (22.64%)	41 (77.36%)	0.686	0.31–1.49	0.342
	G3	6 (40.0%)	9 (60.0%)	1.563	0.50–4.79	0.435
	≥G4	4 (26.67%)	11 (73.33%)	0.852	0.25–2.90	0.799
Single vs. multiple gestations	Single gestation	46 (26.7%)	126 (73.3%)	Reference	-	-
	Multiple gestations	5 (62.5%)	3 (37.5%)	4.56	1.04–19.86	0.043*
Spontaneous vs. artificial conception	Spontaneous	44 (27.1%)	118 (72.9%)	Reference	-	-
	Ovarian induction/IVF	7 (38.8%)	11 (61.2%)	1.706	0.622–4.68	0.299
Associated comorbidities	None	41 (28.25)	104 (71.7%)	Reference	-	-
	Hypothyroid	6 (28.5%)	15 (71.5%)	0.792	0.27–2.30	0.670
	Gestational diabetes mellitus	3 (27.2%)	8 (72.8%)	1.449	0.40–5.21	0.570
	Bronchial asthma	1 (33.3%)	2 (66.7%)	1.268	0.11–14.37	0.848
Indication for LSCS	Fetal distress	30 (24.4%)	93 (75.6%)	Reference	-	-
	CPD/arrest of labor	15 (44.12%)	19 (55.88%)	2.447	1.10–5.40	0.057*
	Previous LSCS	1 (11.1%)	8 (88.9%)	0.387	0.04–3.22	0.381
	Malpresentation	0	6 (100.0%)	1.000	-	-
	Failed induction	5 (62.5%)	3 (37.5%)	5.166	1.16–22.90	0.031*
Duration of labor	<4 hours	2 (11.76%)	14 (82.3%)	Reference	-	-
	4–5 hours	5 (15.15%)	28 (84.8%)	1.339	0.23–7.75	0.744
	6–8 hours	18 (28.57%)	45 (71.4%)	3.000	0.62–14.46	0.171
	>8 hours	26 (38.81%)	42 (62.7%)	4.756	1.00–22.52	0.049

Disease-related factors of preeclampsia did not demonstrate a statistically significant association with hypotension. Early-onset preeclampsia (≤34 weeks) showed a higher proportion of hypotension (18/52 (34.6%)) compared to late-onset preeclampsia (33/128 (25.8%)), although the association was not statistically significant (OR 1.53, p=0.221). Similarly, women with a disease duration greater than one week had a higher incidence of hypotension (16/42 (38.1%)) than those with a shorter duration (35/138 (25.4%)), but this difference did not reach statistical significance (OR 1.79, p=0.124).

Among treatment modalities, patients receiving intravenous labetalol along with oral antihypertensive therapy demonstrated a higher proportion of hypotension (7/13 (53.9%)), whereas comparatively lower rates were observed among women receiving magnesium sulfate (14/49 (28.6%)) and oral labetalol alone (30/118 (25.4%)). However, none of these treatment-related associations were statistically significant (p>0.05) (Table 3).

**Table 3:** Univariate analysis – preeclampsia disease factors for association with risk of hypotension after spinal anesthesia (N=180) *Variables with a p-value less than 0.20 in the univariate analysis will be taken for multivariate analysis for association with hypotension. SBP: Systolic blood pressure; OR: Odds ratio; CI: Confidence interval.

Characteristics		SBP < 100 n (%) n = 51	SBP ≥100 n (%) n = 129	Odds ratio (OR)	95% Confidence interval (CI)	p-value
Gestational age at onset of preeclampsia	≤34 weeks	19 (34.55%)	36 (65.45%)	1.533	0.77–3.04	0.221
	>34 weeks	32 (25.60%)	93 (74.40%)	Reference	-	-
Duration of preeclampsia	<1 week	32 (25.60%)	93 (74.40%)	Reference	-	-
	1–2 weeks	16 (38.10%)	26 (61.90%)	1.788	0.85–3.75	0.124
	>2 weeks	3 (23.08%)	10 (76.92%)	0.871	0.22–3.36	0.842
Drug therapy	Conservative management	3 (50.00%)	3 (50.00%)	Reference	-	-
	Magnesium sulfate (1)	2 (14.29%)	12 (85.7%)	0.166	0.01–1.49	0.109
	Labetalol oral only (2)	11 (15.49%)	60 (84.51%)	0.183	0.03–1.02	0.054
	Labetalol iv bolus with oral labetalol (3)	7 (53.85%)	6 (46.15%)	1.166	0.16–8.09	0.876
	Combination 1 and 2	3 (15.0%)	17 (85.0%)	0.176	0.02–1.32	0.092
	Combination of 1 and 3	25 (44.64%)	31 (55.36%)	0.806	0.14–4.34	0.802

Among perioperative variables, anemia showed a significant association with hypotension following spinal anesthesia. Women with hemoglobin levels of 7-9.9 g/dl demonstrated a higher proportion of hypotension (14/31 (45.2%)) compared to those with hemoglobin >11 g/dl (25/116 (21.6%)) (OR 2.99, p=0.010). Similarly, women with hemoglobin levels between 10 and 10.9 g/dl also had an increased occurrence of hypotension (12/33 (36.4%)), although with lower odds compared to the severely anemic group. Prolonged fasting duration of more than eight hours was strongly associated with hypotension, with 19 out of 34 women (55.9%) developing hypotension compared to 6 out of 37 women (16.2%) with fasting duration less than four hours (OR 6.54, p=0.001). Other laboratory and perioperative parameters, including platelet count, serum creatinine, liver enzyme levels (AST and ALT), urine protein-creatinine ratio, and intraoperative blood loss, did not demonstrate statistically significant associations with hypotension (p>0.05) (Table 4).

**Table 4:** Univariate analysis – perioperative assessment factors for association with risk of hypotension after spinal anesthesia (N=180) *Variables with a p-value less than 0.20 in the univariate analysis will be taken for multivariate analysis for association with hypotension. SBP: Systolic blood pressure; OR: Odds ratio; CI: Confidence interval; AST: Aspartate aminotransferase; ALT: Alanine aminotransferase; PCR: Protein–creatinine ratio; NPO: Nil per oral.

Characteristics		SBP < 100 n (%) n = 51	SBP ≥100 n (%) n = 129	Odds ratio (OR)	95% Confidence interval (CI)	p-value
Hemoglobin	>11 g/dl	25 (21.55%)	91 (78.45%)	Reference	-	-
	10–10.9 g/dl	12 (36.36%)	21 (63.64%)	2.08	0.90–4.79	0.086
	7–9.9 g/dl	14 (45.16%)	17 (54.84%)	2.99	1.30–6.90	0.010*
Platelets	<1 lakhs	1 (20.0%)	4 (80.0%)	0.625	0.068–5.72	0.678
	≥1 lakhs	50 (28.6%)	125 (71.4%)	Reference	-	-
Creatinine	<1 mg/dl	50 (28.5%)	126 (71.5%)	Reference	-	-
	≥1 mg/dl	1 (25.0%)	3 (75.0%)	0.840	0.085–8.268	0.881
AST	≤40 IU/L	46 (28.4%)	116 (71.6%)	Reference	-	-
	>40 IU/L	5 (27.8%)	13 (72.2%)	0.969	0.32–2.87	0.956
ALT	≤40 IU/L	48 (28.3%)	122 (71.7%)	Reference	-	-
	>40 IU/L	3 (30.0%)	7 (70.0%)	1.089	0.27–4.38	0.904
Urine PCR	<5	50 (29.41%)	120 (70.59%)	Reference	-	-
	≥5	1 (10.0%)	9 (90.0%)	0.266	0.03–2.16	0.216
Intraoperative blood loss	≤500 ml	21 (26.92%)	57 (73.08%)	Reference	-	-
	>500 ml	30 (29.41%)	72 (70.59%)	1.13	0.58–2.18	0.714
NPO duration	<4 hours	6 (16.22%)	31 (83.78%)	Reference	-	-
	4–8 hours	26 (23.85%)	83 (76.15%)	1.618	0.60–4.30	0.335
	>8 hours	19 (55.88%)	15 (44.12%)	6.544	2.16–19.77	0.001*

On multivariable analysis, anemia remained an independent predictor of hypotension. Patients with hemoglobin 10-10.9 g/dl had increased odds (AOR 2.45, p=0.044), while those with hemoglobin 7-9.9 g/dl had significantly higher odds (AOR 3.16, p=0.009) compared to those with >11 g/dl. Multiple gestations showed a trend toward increased risk (AOR 4.34), though not statistically significant (p=0.058). Gestational age of onset of preeclampsia was not independently associated with hypotension (p=0.151) (Table 5).

**Table 5:** Multivariable logistic regression analysis of the overall associated factors with hypotension (n = 180) *In multivariate analysis, variables with p-values < 0.05 are statistically significant.

S. No.	Characteristics		Adjusted odds ratio	Confidence interval (CI)	p-value*
1	Single vs. multiple gestations	Single gestation	Reference	-	-
		Multiple gestations	4.34	0.94–19.85	0.058
2	Gestational age of onset of preeclampsia	≤34 weeks	1.712	0.82–3.56	0.151
		>34 weeks	Reference	-	-
3	Hemoglobin	>11 g/dl	Reference	-	-
		10–10.9 g/dl	2.447	1.02–5.84	0.044
		7–9.9 g/dl	3.155	1.33–7.45	0.009

## Discussion

Although the incidence of hypotension following spinal anesthesia is generally lower in preeclamptic women compared to normotensive parturients, its occurrence in this population is clinically significant. Preeclampsia is characterized by widespread endothelial dysfunction and altered vascular responsiveness, which predispose these women to exaggerated hemodynamic fluctuations. A prospective cohort study also reported notable reductions in systolic and mean arterial pressure following spinal anesthesia among preeclamptic women, emphasizing the continued need for close intraoperative monitoring despite the relatively attenuated hypotensive response [9]. Episodes of hypotension in such patients may compromise perfusion to vital organs, including the placenta, brain, and kidneys. Furthermore, the use of vasopressors to treat hypotension can precipitate reactive hypertension, further complicating perioperative management [10-13].

In the present study, hypotension was defined as systolic blood pressure <100 mmHg [14]. Based on this definition, 51 out of 180 preeclamptic women (28.3%) developed hypotension following spinal anesthesia. Analysis of systolic blood pressure trends revealed that the mean SBP was lowest at approximately 20 minutes after spinal anesthesia (mean 112 mmHg, SD 14.60), consistent with the known pharmacodynamic profile of spinal anesthesia, where peak sympathetic blockade occurs within the first 20-30 minutes. The absence of other confounding intraoperative events supports spinal anesthesia as the primary contributor to the observed hypotension.

To the best of our knowledge, limited studies have comprehensively evaluated both obstetric and anesthetic risk factors for hypotension specifically in preeclamptic women. This study, therefore, attempts to address an important gap in the literature. Among demographic and anthropometric factors, age and BMI did not show statistically significant associations with hypotension, although a higher proportion of hypotensive episodes was observed in older women and those with higher BMI. These findings are consistent with previous studies that have reported increased hemodynamic variability in older parturients [15-17]. Similarly, higher gravida status, particularly G3, showed increased odds of hypotension, suggesting a possible cumulative physiological adaptation or vascular remodeling with repeated pregnancies; however, this association was not statistically significant.

A notable finding in this study was the association between multiple gestation and hypotension. Women with twin pregnancies had a significantly higher incidence of hypotension (62.5%) compared to those with singleton pregnancies (26.7%), with an OR of 4.56 (95% CI: 1.04-19.86; p=0.043) on univariate analysis. Although this association lost statistical significance after adjustment (AOR 4.34; p=0.058), the trend remained clinically relevant. The increased risk in multiple gestation may be explained by exaggerated aortocaval compression due to increased uterine size, as well as the higher likelihood of assisted reproductive techniques and advanced maternal age in this group. To date, there is limited literature exploring this relationship in preeclamptic populations, making this finding an important contribution.

The indication for cesarean section also demonstrated variability in hypotension risk. Women undergoing lower segment cesarean section (LSCS) for failed induction had a higher incidence of hypotension (62.5%; OR 5.16; p=0.031), while those with cephalopelvic disproportion or arrest of labor showed increased odds (OR 2.44), though not statistically significant. These findings may reflect prolonged labor, stress, and altered intravascular volume status prior to anesthesia as contributing factors.

Disease-related factors of preeclampsia, including gestational age at onset and duration of disease, did not show significant associations with hypotension. Early-onset preeclampsia (≤34 weeks) showed higher odds (OR 1.53), which persisted after adjustment (AOR 1.71), suggesting a possible trend toward increased hemodynamic instability in severe disease; however, statistical significance was not achieved. Similarly, duration of disease did not demonstrate a meaningful relationship with hypotension, indicating that disease chronicity alone may not be a reliable predictor. The role of antihypertensive therapy was also explored. Although women receiving combined intravenous and oral labetalol demonstrated higher odds of hypotension, this was not statistically significant. This observation may reflect the underlying severity of hypertension rather than a direct pharmacological effect. The hypothesis that aggressive antihypertensive therapy predisposes to hypotension warrants further investigation.

Among laboratory parameters, anemia emerged as a significant and independent predictor of hypotension. Women with hemoglobin levels of 7-9.9 g/dl had significantly higher odds of hypotension (OR 2.99; p=0.010), which remained significant after adjustment (AOR 3.15; p=0.009). Similarly, moderate anemia (10-10.9 g/dl) was also associated with an increased risk (AOR 2.44; p=0.044). Reduced oxygen-carrying capacity and impaired compensatory mechanisms in anemic patients may contribute to hemodynamic instability following spinal anesthesia. This finding contrasts with previous studies in normotensive women, where no such association was observed [18,19], suggesting that anemia may have a more pronounced impact in the preeclamptic population.

Perioperative factors were also evaluated. Prolonged fasting (>8 hours) showed a strong association with hypotension (OR 6.54; p=0.001), likely due to intravascular volume depletion. Although this did not retain significance in multivariable analysis, the clinical relevance remains important. Other intraoperative factors, such as blood loss, liver function parameters, renal function, and proteinuria, did not demonstrate significant associations. Interestingly, despite the occurrence of hypotension, neonatal outcomes assessed by APGAR scores at one and five minutes were not adversely affected. This suggests that timely recognition and management of hypotension may mitigate fetal compromise.

The study has a few limitations to consider. Due to the COVID-19 pandemic, patient admissions were reduced, and the intended sample size could not be achieved, limiting the statistical power of the study. Maternal outcomes in the postpartum period were not assessed, restricting understanding of the long-term impact of intraoperative hypotension. The single-center design may limit the generalizability of findings to other populations. Another limitation is the operational definition of hypotension used in this study. Hypotension was defined as an absolute systolic blood pressure of <100 mmHg rather than a percentage reduction from baseline blood pressure. Although this definition has been employed in previous obstetric anesthesia studies, it may underestimate the incidence of clinically significant hypotension in women with higher baseline blood pressures, particularly in preeclamptic patients.

The findings of this study highlight anemia and prolonged fasting as modifiable risk factors for hypotension in preeclamptic women undergoing spinal anesthesia. Optimization of hemoglobin levels antenatally and minimizing preoperative fasting duration may reduce the risk of hypotension. This is supported by data from studies indicating that reduced physiological reserve and impaired compensatory mechanisms may contribute to hemodynamic instability after neuraxial blockade [20]. Additionally, heightened vigilance is warranted in women with multiple gestations and those undergoing cesarean section after prolonged labor. These results underscore the need for individualized anesthetic planning and careful hemodynamic monitoring in this high-risk population.

## Conclusions

Hypotension following spinal anesthesia occurred in approximately one-third of preeclamptic women and remains a clinically relevant concern despite its lower incidence compared to normotensive pregnancies. Anemia emerged as an independent predictor, while multiple gestation and prolonged fasting showed important clinical associations. Although disease-specific factors of preeclampsia were not significantly associated, the interplay of obstetric and perioperative variables highlights the multifactorial nature of hypotension in this population. Early identification and correction of modifiable risk factors, along with vigilant intraoperative management, are essential to optimize maternal hemodynamic stability and improve perioperative outcomes.
